# Standing up in multiple sclerosis (SUMS): protocol for a multi-centre randomised controlled trial evaluating the clinical and cost effectiveness of a home-based self-management standing frame programme in people with progressive multiple sclerosis

**DOI:** 10.1186/s12883-016-0581-8

**Published:** 2016-05-05

**Authors:** J. A. Freeman, W. Hendrie, S. Creanor, L. Jarrett, A. Barton, C. Green, J. Marsden, E. Rogers, J. Zajicek

**Affiliations:** Faculty of Health and Human Sciences, School of Health Professions, Plymouth University, Peninsula Allied Health Centre, Derriford Rd, Plymouth, PL6 8BH England; Norwich MS Centre, Alkmaar Way, Norwich, NR6 6BB England; Centre for Health Statistics, Peninsula Schools of Medicine and Dentistry, Room 302, Tamar Science Park, Plymouth, PL68BX England; Mardon Neurorehabilitation Centre, Royal Devon and Exeter NHS Foundation Trust, Wonford Rd, Exeter, EX2 4UD England; Research and Design Service, Peninsula Schools of Medicine and Dentistry, ITTC Building, Tamar Science Park, Plymouth, PL68BX England; University of Exeter Medical School, Health Economics Group, University of Exeter, Veysey Building, Exeter, Devon EX24SG England; School of Medicine, Medical and Biological Sciences, University of St Andrews, North Haugh, St Andrews, Scotland, KY169TF UK

**Keywords:** Progressive Multiple Sclerosis, Standing, Standing frame, Physiotherapy, Self-management, Cost effectiveness, Mobility

## Abstract

**Background:**

Multiple sclerosis (MS) is an incurable, unpredictable but typically progressive neurological condition. It is the most common cause of neurological disability in young adults. Within 15 years of diagnosis, approximately 50 % of affected people are unable to walk unaided, and over time an estimated 25 % depend on a wheelchair. Typically, people with such limited mobility are excluded from clinical trials. Severely impaired people with MS spend much of their day sitting, often with limited ability to change position. In response, secondary complications can occur including: muscle wasting, pain, reduced skin integrity, spasms, limb stiffness, constipation, and associated psychosocial problems such as depression and lowered self-esteem. Effective self-management strategies, which can be implemented relatively easily and cheaply within people’s homes, are needed to improve or maintain mobility and reduce sedentary behaviour. However this is challenging, particularly in the latter stages of disease. Regular supported standing using standing frames is one potential option.

**Methods/Design:**

SUMS is a pragmatic multi-centre randomised controlled trial evaluating use of Oswestry standing frames with blinded outcome assessment and full economic evaluation. Participants will be randomly allocated (1:1) to either a home-based, self-management standing programme (with advice and support) along with their usual care or to usual care alone. Those in the intervention group will be asked to stand for a minimum of 30 min three times weekly over 20 weeks. Each participant will be followed-up at 20 and 36 weeks post baseline. The primary clinical outcome is motor function, assessed using the Amended Motor Club Assessment. The primary economic endpoint is quality-adjusted life years. The secondary outcomes include measures of explanatory physical impairments, key clinical outcomes, and health–related quality of life. An embedded qualitative component will explore participant’s and carer’s experiences of the standing programme.

**Discussion:**

This is the first large scale multi-centre trial to assess the clinical and cost effectiveness of a home based standing frame programme for people who are severely impaired by MS. If demonstrated to be effective and cost-effective, we will use this evidence to develop recommendations for a health service delivery model which could be implemented across the United Kingdom.

**Trial registration:**

ISRCTN69614598

**Date of registration:**

3.2.16 (retrospectively registered)

## Background

Multiple sclerosis (MS) is an incurable, unpredictable but typically progressive, life-long, neurological condition, affecting approximately 100,000 people in the United Kingdom (UK) [1]. It is the most common cause of neurological disability in young adults, with an estimated cost of £1.4 billion/annum to the National Health Service (NHS) and society [2]. Although most people start with a relapsing-remitting disease course, approximately two-thirds move to a progressive phase within eight years, at which point medical interventions are limited. Within 15 years of diagnosis, an estimated 50 % of affected people are unable to walk unaided, and over time 25 % become dependent on a wheelchair [3]. It is, therefore, unsurprising that mobility is a major concern for people with MS and health professionals. Surveys of people with MS consistently rank mobility as their highest priority [4, 5] and most important yet most challenging daily function [6]. Mobility has been correlated negatively with employment status and quality of life [2]. Evidence shows that enhancing physical activity and reducing sedentary behaviour can improve mobility and directly associated complications [7], providing a persuasive argument for ensuring that optimal physical management is a clinical priority.

NICE Guidelines emphasise that mobility spans much more than simply walking [7], including activities necessary for daily functioning: the ability to stand, safely transfer from wheelchair to toilet or to bed, and to move in bed. People with MS who are severely impaired spend much of their day sitting, often with limited ability to change position. In response, secondary complications can occur rapidly, including muscle wasting, pain, reduced skin integrity, spasms, limb stiffness, constipation, and associated psychosocial problems such as depression and lowered self-esteem [8]. These secondary problems can accelerate the loss of independence in daily function, and can impact negatively upon quality of life [4, 9] and self-identity. The clinical significance of these issues is underlined by their consistent prominence in policy documents for long-term neurological conditions [7, 10].

There are also significant economic costs related to increasing immobility and secondary complications since they increase the burden of care, delay rehabilitation and increase healthcare costs. It is estimated that approximately 15 % of people with MS will, at some point in time, develop a pressure sore [11], with treatment costs of a single pressure sore ranging from £1,064 to £24,214 [6]. The mean cost per wheelchair dependent patient is 4–5 times higher than an ambulatory patient [12]. This clearly has significant implications to the health services.

Evidence from studies of a range of neurological conditions, including MS, shows that increasing physical activity by interventions such as physiotherapy, can improve balance and mobility and reduce associated secondary health problems which arise from immobility [7]. However, increasing physical activity is difficult when disability is severe. Physiotherapy typically comprises short intermittent episodes of face-to-face sessions. On-going physiotherapy is rarely possible due to resource restrictions [13, 14]. While group-based exercise programmes are becoming more common, they can be inaccessible, and may have inappropriate content, for people with severe impairments [15]. Effective self-management strategies, which can be implemented relatively easily and cheaply within people’s homes, are needed for severely impaired individuals to improve or maintain their ability to move and reduce their sedentary behaviour. However this is challenging, particularly in the latter stages of disease. Regular supported standing using standing frames is one potential self-management option.

A systematic review investigating effectiveness of supported standing in neurological conditions [8], provided some data to support its acceptability, feasibility and efficacy but highlighted that more robust evidence is required. Of 17 randomised controlled trials (RCTs), only two were of high quality, and only one trial involved people with MS [16], which was a short-term, hospital based programme. Our updated literature search additionally identified one mixed-methods study [17] and a qualitative study [18], but no further RCT’s investigating this aspect of MS rehabilitation management.

Among the work of this research team, a study be Hendrie et al., has provided compelling evidence of the need for a trial of a managed standing frame programme. This study used a mixed-methods approach involving nine in-depth single case studies [17] to provide an in-depth understanding of the experiences of people with MS who participated in the standing programme. Over the course of a year, in addition to a range of objective measures, Hendrie undertook 27 in-depth interviews. Respondents’ stories reveal how the programme enabled them to “reconnect with their body”, “regain lost skills”, and gain a sense of “being in control”. In line with other related research [18] these interviews provided convincing qualitative evidence that this self-management standing programme reinstated a sense of belonging and optimism about the future by restoring important life roles and feelings of normality. A particularly debilitating secondary complication of immobility is stiff/hypertonic muscles. Our laboratory-based studies [19] determined that the duration and magnitude of force required to ease the stiffness in hypertonic lower limb muscles could only be achieved in a supported standing position for more severely impaired people with MS.

The proposed study builds on these existing research strands, asking the question “What is the clinical and cost effectiveness of a home-based self-management standing frame programme in people who are severely impaired with progressive MS?” The aim of this definitive multi-centre randomised controlled study is to investigate the clinical and cost effectiveness of a home-based supported standing programme for people severely impaired with progressive MS.

More specifically, the objectives are to:

1. Assess the clinical effectiveness of the standing frame programme in improving motor function (primary outcome).
2. Assess the clinical effectiveness of the standing frame programme in improving balance, muscle strength, joint and muscle range, painful spasms, respiratory, bladder and bowel function, number of falls and quality of life (secondary outcomes).
3. Establish the cost-effectiveness of the standing frame programme versus usual care.
4. Explore the subjective experience of using a standing frame within the home, from the perspective of both the person with MS and their carer.

## Methods

### Trial design

**Fig. 1:**
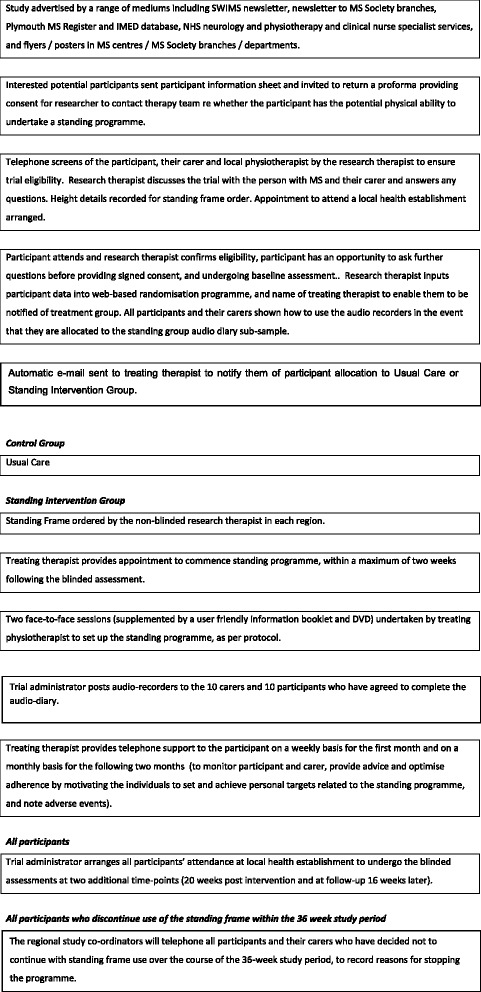
Trial flow chart of participant pathway

This is a pragmatic multi-centre randomised controlled trial with blinded outcome assessment and full economic evaluation. Figure 1 shows the participant pathway.

### Study Setting

Eight healthcare sites will be involved in this multi-centred RCT which is based in two geographical regions of England: Devon/Cornwall and East Anglia. Each site consists of up to seven individual community based therapy teams.

### Participants

#### Sample size

Our primary outcome is the Amended Motor Club Assessment (AMCA) at 16 weeks follow-up. Our primary analysis will utilise analysis of covariance (ANCOVA), comparing AMCA scores at week 36 between allocated groups, adjusting for baseline AMCA score. The trial is powered to detect a between-group difference of nine points which is both plausible and considered clinically relevant [17, 20]. In people with severe MS a score change of 9 points is considered clinically significant, reflecting changes such as allowing a person to balance and lean forwards in sitting to dress themselves or to stand in the shower and wash their hair with two hands.

Estimates of final SD and baseline/follow-up correlation for AMCA are subject to uncertainty and thus we have used conservative 80 % upper confidence limits for both parameters in our calculations. To detect a between-group difference of nine points, with 80 % power and at the 5 % significance level, requires data from 55 participants per group [21].

Allowance is required for people not completing the programme, and for participants not attending the final follow-up assessment. Based on our previous experiences, we have allowed for 20 % loss to follow-up. Therefore the sample size required is 69 participants per group, rounded to a total of 140. An estimated 380 people will need to be screened to achieve the required sample size.

### Inclusion criteria

The study population will comprise individuals diagnosed with primary or secondary progressive MS according to McDonald’s criteria [22].

These participants will:

- be aged >18 years
- be willing and able to consent to participate
- score 6.5–8.0 on the Expanded Disability Status Scale (EDSS), i.e. people who “require bilateral assistance to walk 20 metres or less” to those “restricted to bed or wheelchair”
- be able to get into a standing frame independently or with assistance from one person
- have the agreement of another person (e.g. carer) should assistance be necessary for the standing programme
- be able and willing to accommodate the standing frame in their home
- be willing and able to travel to local assessment centres for blinded outcomes assessment

### Exclusion criteria

Participants will be excluded if they:

- have had any recent changes in disease modifying therapies. More specifically they will be excluded if: they have ever had Alemtuzumab, are within past six months of ceasing Nataluzimab, or are within three months of ceasing any other MS disease modifying drug
- have relapsed/received steroid treatment within the last month
- are currently undertaking a regular standing frame programme (>x1/week), or have done so during the past six months
- have a history of osteoporotic-related fractures
- have co-morbidities which contraindicate standing in the frame (e.g. foot ulceration, uncontrolled epilepsy) or likely to impact on the trial (e.g. chronic jaundice, heart disease, age related multiple co-morbidities)
- currently participating in another clinical trial (rehabilitation or pharmacological)

Identification and recruitment of participants will be via a number of routes which include screening MS databases, identification by healthcare professionals, and advertising via MS support groups and newsletters.

### Randomisation

Participants will be randomised immediately after the baseline assessment. Randomisation, using random sized permuted blocks, will be on a 1:1 basis to receive the intervention plus usual care or usual care only. It will be stratified by region and baseline EDSS (≤7.0 or ≥7.5). The allocation sequence is computer-generated by the local United Kingdom Clinical Research Collaboration Registered Peninsula Clinical Trials Unit (UKCRC PenCTU) (Registration Number 31) in conjunction with an independent statistician who will have no involvement with the final analysis. Following assessment, the blinded assessor will input the details of the participants directly into the randomisation web-site. This will generate an e-mail stating the participant’s allocated treatment group to the regional study co-ordinators and study administrator to notify them of the treatment allocation.

### Blinding

The trial participants and carers are unable to be blinded in this trial due to the nature of the intervention. Similarly, the treating physiotherapists and health care providers are unable to be blinded. However, the outcome assessors will be blinded to participants’ allocated group. All assessments will be undertaken in separate visits independently of delivery of treatment/usual care and away from the participant’s home. Every effort will be made throughout the trial to ensure these assessments are blinded. Upon any interaction with the participant, the blinded assessors will remind participants not to discuss their allocated group, and this will be re-iterated within any written and telephone correspondence.

At each assessment time point, the blinded assessor will be asked to record on a standardised form whether or not they were un-blinded to the group allocation, and if so the reasons for this.

### Interventions

Participants in the intervention group will be asked to stand in an Oswestry standing frame [17] for 30 min three times per week for a total of 16 weeks during a 20 week period. The Oswestry standing frame is a wooden frame, used in clinical practice, which provides support in standing whilst allowing for exercise and controlled movement.

The treating physiotherapist will teach the participant and carer safe and effective use of the standing frame over two face-to-face sessions in the participant’s home (~60 min/session). Participants will also be taught exercises and stretches to undertake in the frame. A detailed user-friendly information leaflet and digital versatile disc (DVD) will be provided to support these sessions. Further support will be provided by weekly telephone contact for four weeks, and then monthly for two months. Telephone calls will focus on facilitating individuals to set and achieve personal targets.

### Standardisation and fidelity of the intervention

Treating physiotherapists from each of the eight healthcare sites will perform the interventions as part of their NHS role; all physiotherapists will implement the intervention in a similar manner.

Use of standing frames is incorporated within undergraduate physiotherapy training and is a recognised core skill for neurological physiotherapists. To standardise and optimise implementation of the intervention, treating therapists, participants and carers will be provided with an information pack. This includes a written template of what is required to be undertaken within each session and a link to the study web-site, which houses a range of resources including a detailed booklet, instructional video with suggested exercise/stretches/balance activities, and evidence based articles on this topic.

All treating therapists will be required to complete a pre-formatted checklist to self-assess their fidelity with the content of the sessions. They will be asked to record any deviations from the protocol on a Protocol Deviations form. In addition, 10 % of the intervention sessions will be assessed in each region by the unblinded regional Principal Investigator who will observe the home-based treatment sessions and independently complete the same fidelity checklist.

### Usual care

This trial will use a usual clinical care control group. A recent MS Society national survey demonstrated that access to physiotherapy services varies throughout the UK/England [14]. Although usual care varies between individuals [1], it rarely involves regular physiotherapy intervention either within the community or hospital [13]. Any intervention is generally limited to a few visits, typically reacting to presenting problems (e.g. practising transfer skills, providing mobility aids) rather than promoting long-term preventative self-management [13]. This national picture reflects the care provided in our study regions. The usual care received (including frequency of physiotherapy intervention as well as any other health/social service interventions and medications) will be recorded within the economic cost effectiveness assessments.

### Data collection and outcome measurements

Standardised, validated clinician-rated and patient self-reported clinical outcomes will be measured at baseline, immediately post intervention (20 weeks) and follow-up (week 36). Any deviations from this will be recorded on a Protocol Deviations form. The longer term follow-up is important to assess maintenance of any observed effect, and determine whether long-term engagement is sustained once support from the treating physiotherapist is withdrawn.

Measures have been selected on the basis of demonstrated reliability and validity in assessing physical impairments, clinical outcomes, quality of life, and economic costs in people with MS.

### Primary outcome measures

The primary clinical outcome is motor function, assessed using the Amended Motor Club Assessment (AMCA) [23]. This rates motor impairment of the lower limb and trunk and key functional movements such as sit-to-stand and standing balance. It was developed specifically for people with MS and has demonstrated validity, reliability and responsiveness [20, 23, 24]

The primary economic endpoint is the quality-adjusted life year (QALY), assessed using the EuroQol five dimensional descriptive system, the EQ-5D-5 L self report measure [25].

### Secondary outcome measures

A. Key clinical outcomes:

1. Bowel and bladder control using the self-report Bladder and Bowel Control Scales [26]
2. Sitting balance using the single item Modified Functional Reach in Sitting [27]
3. Falls frequency through a single yes/no question “Did you fall today?”, recorded on the daily diary
4. Health-related quality-of-life (HRQoL), using the 29 item Multiple Sclerosis Impact Scale (MSIS-29 version 2.0) [28], a disease specific patient-reported outcome measure with a preference-based tariff [29] for use in sensitivity analyses for the Quality Adjusted Life Year (QALY) outcome.

B. Explanatory physical impairments:

1. Knee extensor strength using a portable hand-held dynamometer [30]
2. Length of hip flexors, hamstrings and ankle plantarflexors using manual goniometry [16]
3. Spasm frequency using the Penn Spasm Frequency Scale [31]
4. Respiratory capacity using a hand-held spirometer to record forced expiratory volume at one second [32]

### Measures of intervention adherence

A simple pre-formatted daily diary will be completed by those allocated to the standing frame programme. Either the participant or carer will record adherence with the intervention (frequency, duration, reasons for not standing), and will also have the opportunity to comment on why standing was continued or stopped. Adverse events (including new symptoms and falls) will also be recorded in the diary.

The usual care group will also be asked to complete a daily diary to capture adverse events (including new symptoms and falls).

### Safety monitoring

Participants will be monitored for adverse events via completion of their study daily diaries and during follow-up assessments. The group receiving the intervention will also be monitored during the scheduled telephone calls with their treating physiotherapist. Physiotherapists will be asked to report all adverse events to the research team, whether or not they are thought to be related to the intervention.

### Economic evaluation

The economic evaluation will estimate the cost effectiveness of the standing frame programme plus usual care, versus usual care alone. The primary perspective of the analyses will be that of the NHS and Personal Social Services (i.e. Third Party Payer), with a broader perspective to be considered in sensitivity analyses. Cost effectiveness analyses (CEA) will present an estimate of the incremental cost per unit change in the primary outcome measure (AMCA), but the primary economic analyses will be the incremental cost per QALY gained (over 36 week follow-up). The EQ-5D-5 L will be the primary economic endpoint used to estimate QALYs, applying a UK tariff [33], at final follow-up, with the MSIS-8D [29] used to estimate QALYS based on the MSIS-29, a condition specific measure. Economic analyses will estimate the resource use (e.g. standing frames, physiotherapy sessions, travel, telephone calls, social care assistance) and related cost associated with delivery of the standing frame programme, in addition to usual care, via data collected within the trial.

Data will be collected, via a self-report Resource Use Questionnaire, on participant use of health and social care services (primary, secondary and social care), and on broader aspects of participant and carer-related resource use.

Regression methods will be used to estimate incremental costs and QALYs, adjusting for baseline (cost, QALY) values, with covariates for baseline ACMA and stratification variables (baseline EDSS, geographic regions), and economic analyses will be consistent with the primary statistical analysis plan. The CEA will synthesise cost and outcome data, and explore uncertainty, to present results of the economic evaluation in a policy relevant way.

### Qualitative assessment

A qualitative approach will be used to capture and explore the “real-life” experiences of people’s participation in the standing frame programme. Purposive sampling to achieve maximum variation will be used to request 10 participants and 10 carers to keep audio diaries of their experiences of standing and using the frame. They will be asked to use an audio recorder to record their reflections and experiences on how it feels to stand, changes they are experiencing, plus any comments they wish to make throughout the intervention period. To ensure contemporaneous collection of data they will be asked to record these reflections, if possible, during each stand or as near to the completed standing period as possible.

### Statistical analysis

A full statistical analysis plan will be developed and approved by an independent statistician, prior to final database-lock. The primary statistical analysis will utilise analysis of covariance (ANCOVA), comparing AMCA scores at week 36 between allocated groups, adjusting for baseline AMCA score and the two stratification variables (baseline EDSS category and geographical region). The primary comparative analyses of all outcome measures will be on the basis of intention-to-treat (adverse events will be presented per protocol if different). Between-group differences will be presented with 95 % confidence intervals wherever possible, with the significance level for hypothesis testing set at 5 %, unless otherwise stated.

Secondary outcomes will be compared between groups in a similar way to that for the primary outcome. Comparisons of interest will be presented with 95 % confidence intervals for both unadjusted and adjusted between-group comparisons.

### Subgroup analyses

Exploratory analyses of the possible interaction between baseline EDSS and allocated group will be undertaken, to explore whether any difference between the allocated groups is modified by baseline EDSS category (≤7.0 vs ≥7.5).

### Qualitative analysis

Participants’ stories will be gathered, analysed and reported using narrative methodology [34]. The texts from audio recordings will be left whole, crafted into a story, and not fractured into themes, as an attempt to capture the immediate impact of standing, and to form a chronological record of the person’s experience. This promotes a contextual and holistic account; providing stories that are intended to resonate with people with MS, their families and professionals. By reading these stories the aim is to encourage the reader to reflect on their own situation gaining insight into their own experience as a person standing and living with MS, assisting a person to stand or as a professional.

### Data management, audit and monitoring

Data will be recorded on study specific data collection forms by the research therapists. Completed forms will be entered onto a password-protected customised database, developed by the Peninsula CTU. All data will be double entered and compared for discrepancies. Discrepant data will be verified using the original paper data sheets.

Participants’ anonymity will be maintained on all documents. Data will be collected and stored in accordance with the Data Protection Act 1998, and will be accessible for the purposes of monitoring, auditing, or at the request of the regulatory agency.

### Trial management committees

There are two trial management groups involved in the set up and management of this trial: the Trial Management Group (TMG) and the Trial Steering Committee (TCS).

The TMG comprises 10 individuals involved in the study design and protocol development. The group will meet approximately monthly to oversee the general management of the day to day running of the trial and release the trial results and publications. Closely involved with the sponsors, most members are co-investigators on this study. In contrast the decisions made by the TSC are independent of the sponsors and investigators. The TSC, which meets annually, comprises eight individuals, with majority independent representation (chair, external statistician, member of MS Society and two independent lay members. In addition the chair of the TSC will receive a quarterly update of the adverse events, and a telephone conference / additional face-to-face meeting will be instigated by the Chair or the Chief Investigator should any issues need to be discussed.

The role of the TSC is to oversee the conduct of the trial, including for instance monitoring adverse events, recruitment and attrition rates, the project timeline and finances.

### Ethics

The trial will be conducted in accordance with the ethical principles that have their origin in the Declaration of Helsinki, 1996; the principles of Good Clinical Practice, and the Department of Health Research Governance Framework for Health and Social Care, 2005. The study protocol, participant information and enrolment procedures were assessed and approved through the National Research Ethics Scheme (NRES Committee South West – Frenchay, REC ref no. 15/SW/0088) and management permissions gained from the Research and Development Departments of the eight participating centres in accordance with NHS research governance arrangements. Any amendments to the protocol will be reported to, and approved via NRES.

### Dissemination plan

We will target users, clinicians, researchers, organisations developing clinical guidelines and NHS decision makers. On completion of the trial, the full study report will be accessible on the study web-site page, as will the full protocol. This protocol (Version 3.0, dated 5.2.2015) has been published in line with SPRIT Guidelines [35]. Similarly CONSORT (Consolidated Standards of Reporting Trials) [36] and The Template for Intervention Description and Replication (TiDIER) Guidelines [37] will be reviewed prior to submitting future publications of the trial results to high quality journals. Authorship of intended articles will be by the study team; professional writers will not be used. Results will be presented at national and international conferences to ensure dissemination to academics and those responsible for service delivery. In addition results will be disseminated through the newsletters of MS organisations and via talks to their local support groups. If proven effective, the training materials (treatment manual, instructional video recordings, case scenarios) will be made freely available via the study website, with the aim of optimising roll out. Extracts from the audio recordings will also be used as educational and decision making tools for use by both people with MS and therapists involved in their care. All participants, who consent to receiving notifications, will be notified in writing of trial outcomes.

## Discussion

Preliminary evidence from two small-scale studies suggests that the use of standing frames in the home may improve physical and psychological well-being in people with MS [16, 17]. This protocol describes the first large-scale trial to evaluate the use of the standing frame in this way. It is important to determine definitively whether such a programme is clinically and cost effective so that people affected by MS, clinicians, commissioners and policy makers can make informed evidence-based decisions regarding the use of this intervention.

Some of the choices in the study design warrant discussion. First we discuss why our inclusion criteria were confined to those individuals with progressive disease. This is for two main reasons: the lack of disease-modifying drugs for progressive MS which means that management is aimed solely at minimising symptoms and, if possible, improving function; and the paucity of studies devoted to improving mobility in people with progressive MS [38]. As a consequence there are few proven interventions for these people. By testing the use of standing frames in a multi-centre randomised controlled trial, we may be able to identify a successful and cost effective intervention. In doing so, the results have the potential to change clinical practice and optimise supported self-management within a population that receive little attention, yet are severely affected by their condition.

The second relevant issue is the stratification by region and baseline disability level (as determined by the EDSS ≤7.0 or ≥7.5). We stratified by region (South West England versus East Anglia) because of possible differences in terms of usual care provided. Stratification by disability level was included because of the potential differences in ability of participants to adhere to the intervention, and respond to it. We hypothesise that those with higher levels of disability will have lower levels of adherence and be at higher risk of drop out of the study. Furthermore, more severely impaired participants may utilise greater levels of health and social service provision which may bias the cost effectiveness and cost-utility analyses.

Third, the dose (frequency and intensity) of the self-management standing intervention and the time frame over which this was increased was informed by a combination of scientific and anecdotal evidence. The standing duration of 20 weeks factors in up to four weeks for individuals to become gradually re-accustomed to an upright position and to steadily achieve the desired intensity of standing (three times a week for 30 min per session over 16 weeks) which was based on previous studies [16, 17]. In doing so, we hope that it will minimise fatigue, and allow for time when the participant is unable to use the frame (illness, holidays, etc.), as highlighted by our user discussion groups.

Fourth, a practical issue that we considered was which of the various commercially available standing frames we would utilise for this study. The Oswestry Standing Frame was chosen because: (a) it is commonly used within both the National Health Service, the charitable sector (e.g. MS Centres), and within undergraduate physiotherapy teaching, hence minimising the training requirements for clinicians; (b) it is both durable and recyclable, and hence can be recirculated for use within the health service thereby enhancing sustainability; (c) it is relatively lightweight (the frame can easily be pushed across a floor by one person) which makes it easy to move into a position for standing in the home and then returned to a storage part of the room when not in use; (d) it is used within current clinical practice in a range of conditions (e.g. stroke, spinal cord injury, cerebral palsy) and hence, should the results of this trial be negative, then it could continue to be recycled for use within the health service; (e) it is one of the cheapest frames on the market, and finally (e) it is wooden and hence perceived by some patients as “more like a piece of furniture”, which is an important consideration given that these frames will be used within people’s homes.

### Trial status

Recruitment started in September 2015 and is ongoing (49 participants recruited as of 21st March 2016).

### Ethics approval and consent to participate

The study protocol, participant information and enrolment procedures were assessed and approved through the National Research Ethics Scheme (NRES Committee South West – Frenchay, REC ref no. 15/SW/0088) and management permissions gained from the Research and Development Departments of the eight participating centres in accordance with NHS research governance arrangements. Any amendments to the protocol will be reported to, and approved via NRES.

### Availability of data and material

Data generated as a result of this study, and on which the conclusions of future manuscripts will be relied, will be presented in the main paper or additional supporting files.

## Abbreviations

AMCA: Amended Motor Club Assessment
ANCOVA: analysis of covariance
CEA: cost effectiveness analyses
CONSORT: Consolidated Standards of Reporting Trials
CTU: Clinical Trials Unit
DVD: digital versatile disc
SUMS: Standing Up in Multiple Sclerosis
EDSS: Expanded Disability Status Scale
HRQoL: Health-related quality-of-life
MSIS: Multiple Sclerosis Impact Scale
NHS: national health service
NRES: National Research Ethics Scheme
QALY: quality adjusted life year
RCTs: randomised controlled trials
SUMS: multiple sclerosis
TCS: trial steering committee
TiDIER: Template for Intervention Description and Replication
TMG: trial management group
UK: United Kingdom
UKCRC: United Kingdom clinical research collaboration
